# The New and the Old: Platform Cross-Validation of Immunoaffinity MASS Spectrometry versus ELISA for PromarkerD, a Predictive Test for Diabetic Kidney Disease

**DOI:** 10.3390/proteomes8040031

**Published:** 2020-10-28

**Authors:** Scott Bringans, Kirsten Peters, Tammy Casey, Jason Ito, Richard Lipscombe

**Affiliations:** Proteomics International, Perth, WA 6009, Australia; kirsten@proteomics.com.au (K.P.); tammy@proteomics.com.au (T.C.); jason@proteomics.com.au (J.I.); richard@proteomics.com.au (R.L.)

**Keywords:** biomarkers, assay development, diabetic kidney disease, immunoaffinity, multiplex, targeted mass spectrometry, ELISA, MRM, antibodies

## Abstract

PromarkerD is a proteomics derived test for predicting diabetic kidney disease that measures the concentrations of three plasma protein biomarkers, APOA4, CD5L and IBP3. Antibodies against these proteins were developed and applied to a multiplexed immunoaffinity capture mass spectrometry assay. In parallel, and facilitating current clinical laboratory workflows, a standard ELISA was also developed to measure each protein. The performance characteristics of the two technology platforms were compared using a cohort of 100 samples, with PromarkerD test scores demonstrating a high correlation (R = 0.97). These technologies illustrate the potential for large scale, high throughput clinical applications of proteomics now and into the future.

## 1. Introduction

The application of proteomics technologies for the measurement of protein concentrations has a long history including the use of optical density measurements, SDS-PAGE, amino acid analysis and a range of mass spectrometry techniques. This simple application of technology has increasing importance as research programmes continue producing novel and often complex panels of biomarkers for disease detection and monitoring. As proteomic technology has advanced so too have the methods by which proteomics can be applied to protein concentration measurement. The requirements to go deeper into the proteome to measure that elusive biomarker have produced an arms race between the expanding technology capabilities and the increased sensitivity and specificity demanded of novel biomarker detection. This manuscript describes methods both new and old that make up a repertoire of techniques that can be used to address the fundamental principles of accurate, reproducible protein measurement for specific diseases. 

Chronic kidney disease (CKD) affects one in three adults with diabetes, accounting for 40,000 deaths and $100 billion (USD) in healthcare spending [1]. The current usual-care tests are the urinary albumin:creatinine ratio (ACR) and the blood estimated glomerular filtration rate (eGFR). While these tests can independently determine a person’s kidney function, they can provide conflicting diagnoses and their predictive power to determine a patient’s disease progression is minimal [2].

A proteomics biomarker discovery workflow was undertaken [3] to develop the PromarkerD test that can successfully predict four years in advance that a patient will develop chronic kidney disease (CKD) [4,5]. A panel of three protein biomarker concentrations (Apolipoprotein A4 (APOA4), CD5 antigen-like (CD5L) and Insulin-like growth factor-binding protein 3 (IBP3)) and three clinical variables (age, hdl-cholesterol and eGFR) are combined in a predictive algorithm [5]. The original assay workflow involved firstly immunodepletion of the top 14 plasma proteins, and then diafiltration, reduction, alkylation, digestion and targeted mass spectrometry [3]. This process was suited to a research test that could validate the utility of the biomarker and clinical measurements but ultimately was not sufficient for current clinical application where a more rapid and cost-effective assay was required. 

To provide a higher-throughput and more robust test, two complementary but distinct new methods were developed. An immunoaffinity mass spectrometry assay (IAMS) and an enzyme-linked immunosorbent assay (ELISA). Both were derived from the development of antibodies against the three biomarker proteins. 

The IAMS method uses a bead-bound antibody mixture to capture all three biomarkers from a plasma sample before washing, reduction, alkylation and digestion on the bead. The resulting peptide solution is then analysed by LCMSMS and with the use of calibration protein standards, a concentration of each biomarker is determined. The advantages of this method are the selectivity and hence cleanliness and sensitivity achievable with an antibody capture step and the subsequent multiplexing capability of multiple reaction monitoring (MRM). Independent of this study, the IAMS method was shown to be reproducible and provide equivalent results to the original immunodepletion MS method [6].

The ELISA technology, in use for close to 50 years [7,8], utilises pairs of antibodies developed against recombinant forms of the biomarker proteins. Each plasma biomarker concentration is derived from a standard curve of the recombinant protein with each biomarker measured in a separate well from a distinct dilution of the original plasma sample. The advantages of an ELISA system for clinical biomarker measurements are well known [9,10] and at present is the default method for measuring protein concentrations in many clinical laboratories around the world.

Collectively the IAMS and ELISA methods were tested for aspects of assay reproducibility, robustness and stability before comparison with a cohort of 100 plasma samples on each platform. These newly developed PromarkerD assays represent different strategies and workflows that are applicable in achieving high-throughput, robust, reproducible measurements of protein biomarkers that can be applied to any potential biomarker of any disease. 

## 2. Materials and Methods 

### 2.1. Reagents

Chemicals were from Sigma unless otherwise stated. NP-40, Tris (2-carboxyethyl) phosphine hydrochloride (TCEP) from Thermofisher; Iodoacetamide (IAM) from Astral Scientific. Synthetic isotopically labelled peptides (AQUA, Sigma, St. Louis, MO, USA) were LEPYADQL-R(13C6,15N4) from APOA4 protein, LVGGDNL-C(CAM)-SG-R(13C615N4) from CD5L protein, and FLNVLSP-R(13C6,15N4) from IBP3 protein. A standard reference plasma was created by combining EDTA plasma from three healthy volunteers before aliquoting and storage at −80 °C.

### 2.2. Clinical Samples

All clinical plasma samples were provided by the Fremantle Diabetes Study (FDS), a longitudinal observational cohort [11]. EDTA (Ethylenediaminetetraacetic acid) plasma was collected from all patients after an overnight fast and stored at −80 °C until required. The FDS protocol was approved by the South Metropolitan Area Health Service Human Research Ethics Committee. All subjects gave informed consent before participation. The clinical characteristics of the 100-person cohort from FDS are shown in Supplementary Table S1. These participants were similar in age, gender, BMI, diabetes duration, fasting plasma glucose and glycated hemoglobin (HbA1c) to those in the larger cohort used for the development and validation of PromarkerD (all *p* > 0.05) [5].

### 2.3. Antibody Production

A monoclonal antibody targeting the biomarker APOA4 was developed by the Monash Antibody Technologies Facility (MATF, Monash University, Melbourne, Australia) and the second antibody targeting APOA4 was developed by CDI laboratories (Mayaguez, PR, USA). Both pairs of monoclonal antibodies targeting CD5L and IBP3 were developed by CDI laboratories (Mayaguez, PR, USA). The hybridoma cell lines for all antibodies were used for the production of purified antibodies by the Monoclonal Antibody Facility of the Harry Perkins Institute of Medical Research (Perth, Australia), tested for specificity and provided for use.

### 2.4. Bead-Antibody Production

Batches of magnetic bead-antibody conjugates were made from Dynabeads^®^ (M-270 Epoxy beads, Thermofisher, Waltham, MA, USA) according to the manufacturer’s instructions at a ratio of 50:1 beads:antibody. The bead-antibody conjugate batches (“Ab-beads”) were quality control tested and stored in PBS at a concentration of beads at 10 mg/mL at 2–8 °C until use. Long term stability testing indicates a shelf life of at least 5 months at 2–8 °C (data not shown).

### 2.5. Standards and Controls for IAMS Method

A calibrator standard was prepared by combining the recombinant protein biomarkers APOA4, CD5L and IBP3 (Sino Biologicals, Beijing, China) in phosphate-buffered saline (PBS) with human serum albumin (HSA, 5%, Sigma) as a carrier protein, at concentrations of 18.3, 0.686 and 0.178 µg/mL respectively. Once processed alongside plasma samples they represented effective plasma concentrations of 91.5, 3.45 and 0.892 µg/mL. The synthetic isotopically labelled peptides were used for signal normalisation. The standard reference plasma (stored at −80 °C) was processed (4 separate aliquots) with every batch of samples to allow the monitoring of assay performance and quality control (QC) measurements. The acceptance criteria for a batch of samples (96 well plate) was that the standard reference plasma biomarker concentrations interpolated from the calibrator standard were within 2 standard deviations of the rolling averages of the biomarker concentrations from all the batches for which data had been acquired. 

### 2.6. Sample Processing for IAMS Method

Equal volumes of “Ab-beads” were pooled from the three individual stock solutions, then the liquid removed using a magnet to hold the beads. The beads were then resuspended in a volume of PBS to provide 150 µL volume of beads in each well corresponding to 120 µg of each antibody-bead conjugate per well. The following steps were carried out with a robotic handling system (Janus, Perkin Elmer, Waltham, MA, USA) unless otherwise stated. The Ab-beads were transferred from a trough to a 96 well plate (2 mL round bottom, Thermofisher). The calibrator standard (*N* = 4 replicates, 50 µL), the reference plasma (*N* = 4 replicates, 10 µL) and the samples (10 µL) were added to the plate along with blanks (PBS, 50 µL) and a double blank (200 µL PBS, no Ab-beads). All plasma samples had an additional 40 µL of PBS added to make all final volumes to 200 µL. The plate was incubated at 37 °C for 90 min with intermittent shaking (Thermomixer, Eppendorf, Hamburg, Germany) to keep beads suspended in solution. The liquid was removed with the plate on the magnet and the beads washed with 800 µL of 50 mM triethylammonium bicarbonate (TEAB), 150 mM NaCl, 0.1% NP-40 and then a further wash with 800 µL of 50 mM TEAB. After removing the liquid, the beads were resuspended in 56 µL of 50 mM TEAB, 5 mM TCEP containing 400 fmoles of each of three synthetic 13C15N labelled peptides (one for each target protein) and incubated at 55 °C for 20 min (Thermomixer, Eppendorf). To each well was added 6 µL of 100 mM IAM and incubated at room temperature (RT) in the dark for 20 min. Sequencing grade trypsin (Sigma, catalog no. 1418475001) was then added to each well (10 µL of 0.05 µg/µL in Milli-Q water) and incubated at 37 °C for 16 hrs. The solution was transferred into a clean 96 well plate (300 µL V bottom, Greiner) for LCMSMS analysis.

### 2.7. Targeted (MRM) LCMSMS Analysis for IAMS Method

The system used was a Shimadzu Prominence HPLC system with Loading pump (40 µL/min) to load the sample onto a trap column and two Nano pumps operating at a combined 5 µL/min to provide the analytical gradient. The column was kept at 40 °C and the 5 µL/min flow was directed to a QTRAP 5500 mass spectrometer (Sciex, Framingham, MA, USA) equipped with a 50-micron electrode and grounding unit (Sciex) for the Turbo-V ion source. From the V bottom plate, 25 µL of each sample for analysis was injected onto a MicroLC Guard Column C18 (Sciex) flowing isocratically at 2% (*v*/*v*) Acetonitrile, 0.1% (*v*/*v*) formic acid at 40 µL/min for 2 min. The flow was then directed from the microflow pumps (5 µL/min) through the guard column into a ChromXP C18, 3 µm 120 Å 300 micron ID, 5 cm column (Sciex) with a gradient of 10–40% acetonitrile, 0.1% formic acid over 2 min. The flow was ramped to 98% acetonitrile over 0.2 min, held at that concentration for 0.2 min before returning to the starting conditions of 10% acetonitrile, 0.1% formic acid over 0.1 min. The total runtime was 8 min. The mass spectrometry settings were as follows: Source Temp 250 °C; IonSpray Voltage 5500; Curtain Gas 25; Collision Gas Medium; GS1 and GS2 25; Entrance Potential 10; Collision Cell Exit Potential 14; Q1 Resolution Low; Q3 Resolution Low. The transition settings are shown in Supplementary Table S2.

### 2.8. Data Analysis for IAMS Method

Raw data files were imported into Skyline (v 3.6, MacCoss Lab Software, University of Washington, Seattle, WA, USA) and peaks integrated to provide peak areas for each of the peptides and their corresponding labelled version with a signal to noise (S/N) >5 for all peaks based on the Total Area and Total Background values provided by Skyline. The calibrator replicate ratios of unlabelled:labelled peptide peak area for each biomarker protein were averaged and the known concentration of the calibrator protein was used to determine the concentrations of each protein in unknown samples. In this way, the labelled peptide is used to normalise the signal across all samples analysed while the calibrator protein standards provide the concentrations.

### 2.9. Linearity, Detection Range, Stability, Reproducibility and Precision Testing

Linearity and detection range of the assay was demonstrated with a dilution series of the recombinant biomarker proteins in PBS and 5% HSA that was processed and analysed as for plasma samples.

To assess sample stability to freeze/thaw and other temperature variations the following experiments were carried out.

1. Two duplicates of 3 independent plasma samples (stored at −80 °C) were defrosted to 4 °C and then *N* = 3 samples were processed at 1 hour versus the duplicate *N* = 3 samples left at 4 °C for 24 h before processing.
2. Two duplicates of 3 independent plasma samples were defrosted with *N* = 3 kept at RT for 1 h before processing versus the duplicate *N* = 3 samples left for 24 h at RT before processing.
3. Three independent plasma samples were defrosted versus a set of *N* = 3 replicates that had two more additional freeze-thaw cycles (at least 1 hr frozen at −80 °C before thaw) performed on them before processing.
4. Previously injected replicates of processed plasma (*N* = 86) were left in the HPLC autosampler for a further 24 h after their initial analyses before being re-injected.
5. The precision testing was achieved with *N* = 4 replicates of a reference plasma processed in a single batch for an intra-day comparison and then this was repeated over twenty separate days for an inter-day comparison.

### 2.10. ELISA 

The ELISA assay for the detection of APOA4, CD5L and IBP3 was developed by TGR BioSciences (Adelaide, SA, Australia). Antibodies were supplied as described above under “Antibody production”. Briefly, the ELISA uses the CaptSure™ technology (TGR BioSciences, Adelaide, SA, Australia) based on the principle of traditional sandwich format, but with a more efficient, faster, and simpler assay protocol than standard sandwich immunoassay. The analyte and both chemically tagged antibodies are added at the same time to the microplate that is pre-coated with the CaptsureTM reagent (TGR BioSciences) to immobilise the tagged antibody/biomarker complex. After a 1 hr incubation period, unbound assay reagents and analytes are washed away, and immuno-complexes are detected at an optical density of 450 nm. A 7-point standard curve for each biomarker covers the range of detection in plasma with different dilutions of the plasma required for each biomarker detection. Low and High QCs of recombinant protein are present on the plate to ensure assay integrity.

### 2.11. Cross-Platform Comparisons

A cohort of 100 patient samples from the FDS cohort were analysed by both the IAMS method and the developed ELISA. The biomarker concentrations from each method were then compared using Bland Altman plot analysis to determine the agreement between the two assays [12]. The mean bias between the two methods for each biomarker was determined from the Bland Altman difference plot. Any bias observed was then adjusted by applying the mean difference to the biomarker concentrations measured by ELISA. A hypothesis test for equality (Student’s t-test) was used to assess whether there was a statistical difference between concentrations measured by the two methods. The null hypothesis was that the bias is equal to zero (i.e., there is no difference between the two methods), against the alternative hypothesis that it is not equal to zero. Significant test *p*-values resulted in rejecting the null hypothesis and concluding that the bias is different from zero. The PromarkerD risk scores were calculated for each patient sample from each analysis method, using the adjusted ELISA concentrations. The two PromarkerD scores were then compared by the scatter plot and the correlation between the two methods assessed. An allowable difference of 5% was used to assess the number of subjects with larger differences between the methods. The acceptable percentage of subjects within the 5% difference was set at >90% of the cohort.

## 3. Results

Antibodies against the three biomarkers used in the PromarkerD test were produced to use them to develop an IAMS method and a more traditional ELISA test. The IAMS assay required a single antibody coupled to a magnetic bead while the ELISA used a pair of antibodies for each biomarker.

### 3.1. Immunoaffinity LCMSMS Assay (IAMS)

**Figure 1:**
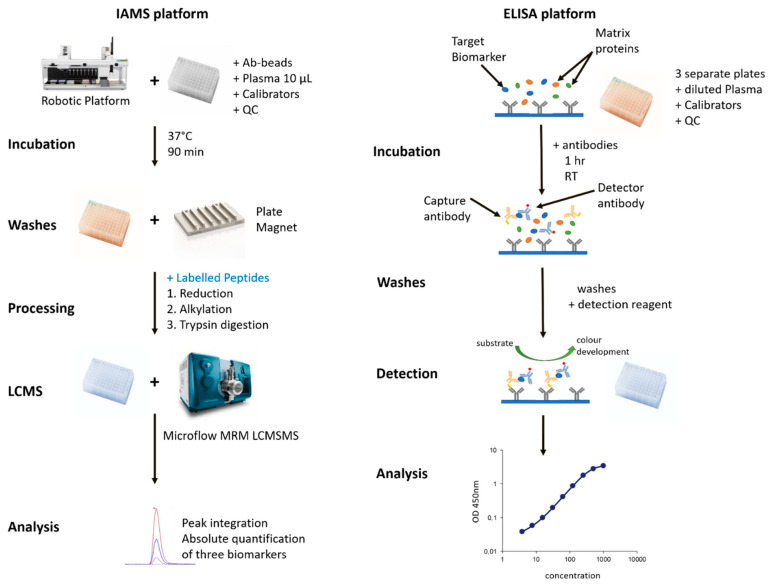
Comparison of the immunoaffinity mass spectrometry assay (IAMS) and ELISA platforms for analysis of three PromarkerD protein biomarkers from plasma.

Antibodies against each biomarker were separately coupled to activated beads and firstly tested for their ability to bind purified recombinant protein and then the native proteins in plasma. After optimisation to produce a reproducible signal response the assay was developed in a 96 well format that allowed for the implementation of automated processing on a robotic handling platform for liquid handling steps. The use of magnetic beads enabled the easy removal of solutions with no meaningful loss of bead material. The original immunodepletion method used a nano LCMS (400 nL/min) with a 90 min run length. In contrast, the IAMS LCMS was optimised to an 8 min run using a flow rate of 5 µL/min into the ion source with a source needle of 50-micron internal diameter to provide spray stability at this flow rate. This method is shown in Figure 1 with a comparison to the ELISA method. 

The linearity, limits of detection (LOD), limits of quantification (LOQ) and range of the assay were provided from a six-point dilution series of the three biomarkers processed by the assay [6]. The plasma working range and the LOD are shown in Table 1 (alongside equivalent values from the ELISA) with the R^2^ value for all three biomarkers >0.98. These quantification ranges covered the range of concentrations found in plasma samples in general using each method.

The robustness of the PromarkerD IAMS assay was demonstrated by temperature stability testing and intra/inter-assay comparison from biomarker measurements of a reference plasma. The temperature stability of the biomarker proteins was assessed using replicates of 3 independent plasma samples that were left for 1 h or 24 h at 4 °C or RT before processing and analysis. Similarly, multiple freeze-thaw cycles were tested on replicates of the 3 plasma samples as well as the stability of processed samples for 24 h at 4 °C to simulate delays in the injection of processed samples. For ligand binding assays (such as this IAMS method) the acceptance criteria, as defined by the FDA bioanalytical guidelines [13] is an accuracy within 20% of the nominal value and a precision CV% of <20% for the measurement of each biomarker. The results indicate that all biomarker comparisons between RT or 4 °C, between 1 h or 24 h, and between up to three freeze/thaw cycles, met these criteria [6]. 

Intra-assay (*N* = 4) and inter-assay (*N* = 20) comparisons of the biomarker concentrations were also demonstrated on the same reference plasma and shown in Table 2 as well as equivalent ELISA values. 

The variability of both intra-assay or inter-assay measurements across 20 separate assays spanning a timeframe of two months had CVs < 11%. Collectively the stability and inter/intraday assay results demonstrate the robustness and reproducibility of measurement using the IAMS methodology. 

### 3.2. ELISA Development and Testing

The ELISA assay was developed by TGR laboratories (Adelaide, Australia) from antibodies supplied to them. The ELISA utilised the Captsure^TM^ technology, a simple, one-step procedure, making it faster and easier to carry out than standard sandwich immunoassays. The basic method is compared to the IAMS method in Figure 1. 

The concentration measurements were based on interpolation from a 7-point dilution series of each of the three recombinant protein standards with the R^2^ value of the fitted equation for each standard curve ≥0.99. Each biomarker had a separate plasma dilution factor and hence were run in separate wells. The working ranges and dilutions were shown in Table 1 with the IAMS data. 

The stability of the plasma protein biomarkers was tested over multiple freeze-thaw cycles with five different plasma samples. All three biomarkers demonstrated robustness for up to 3 freeze-thaw cycles with average CV’s across the 5 samples and 4 measurements (0, 1×, 2×, 3× freeze thaws) to be 9.0%, 3.5% and 4.3% for APOA4, CD5L and IBP3 respectively.

Intra-assay precision was determined by analysing up to 3 biological replicates of plasma in the same assay on the same day. Inter-assay precision was determined by analysing the average of up to 3 biological replicates of plasma in 3 different assays. The precision of the ELISA in measuring the three biomarkers was shown in Table 2 with equivalent IAMS data.

### 3.3. IAMS vs ELISA Platform Comparison

**Figure 2:**
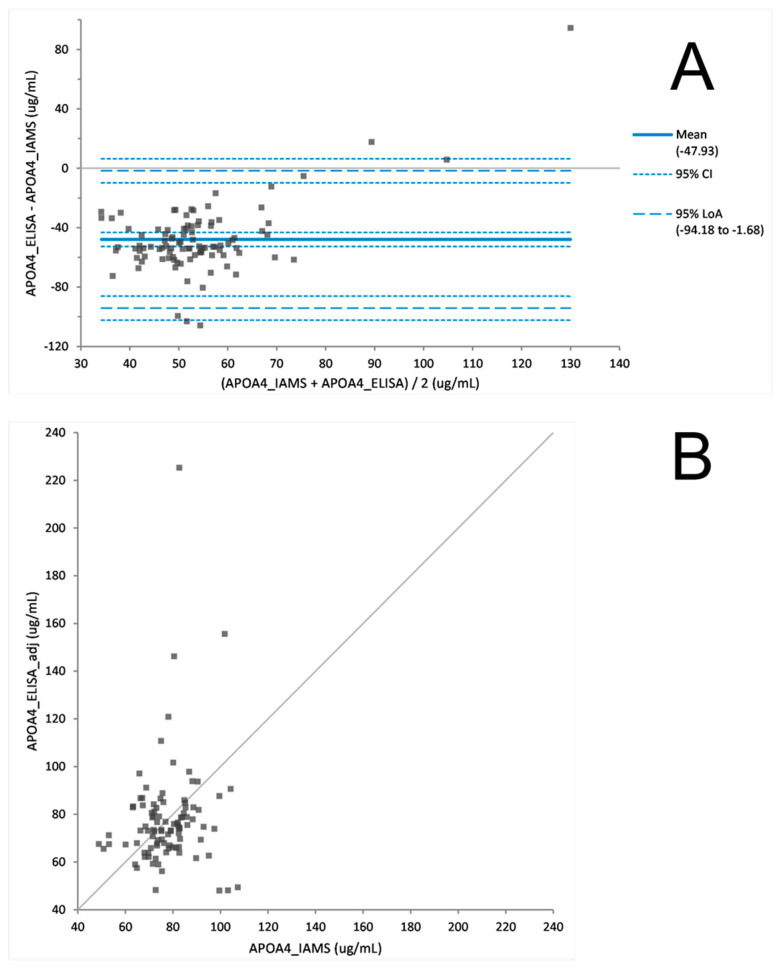
Difference plot (**A**) before Bland Altman correction comparing APOA4 concentrations derived from IAMS and ELISA platforms. Scatter plot (**B**) after adjusting ELISA concentration values with a Bland Altman analysis (adj = adjustment of +47.93 µg/mL to the ELISA value).

**Figure 3:**
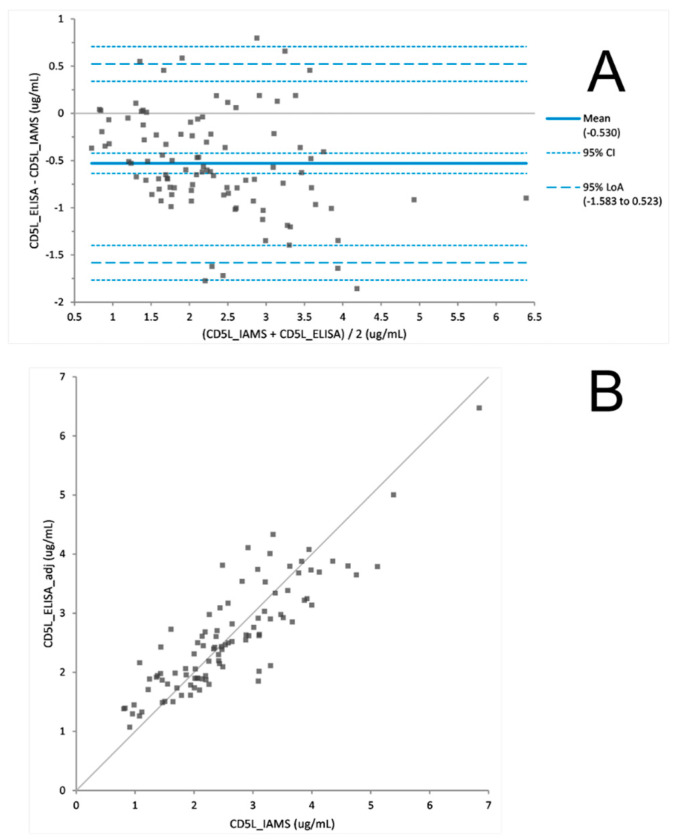
Difference plot (**A**) before Bland Altman correction comparing CD5L concentrations derived from IAMS and ELISA platforms. Scatter plot (**B**) after adjusting ELISA concentration values with a Bland Altman analysis. (adj = adjustment of +0.53 µg/mL to the ELISA value).

**Figure 4:**
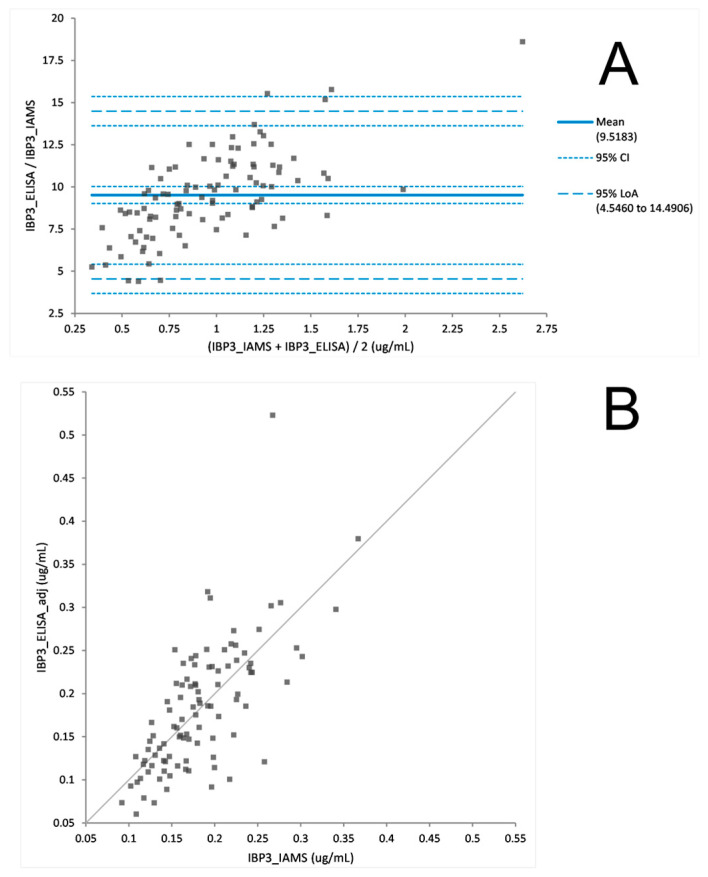
Difference plot (**A**) before Bland Altman correction comparing IBP3 concentrations derived from IAMS and ELISA platforms. Scatter plot (**B**) after adjusting ELISA concentration values with a Bland Altman analysis. (adj = adjustment of division by 9.52 µg/mL to the ELISA value).

**Figure 5:**
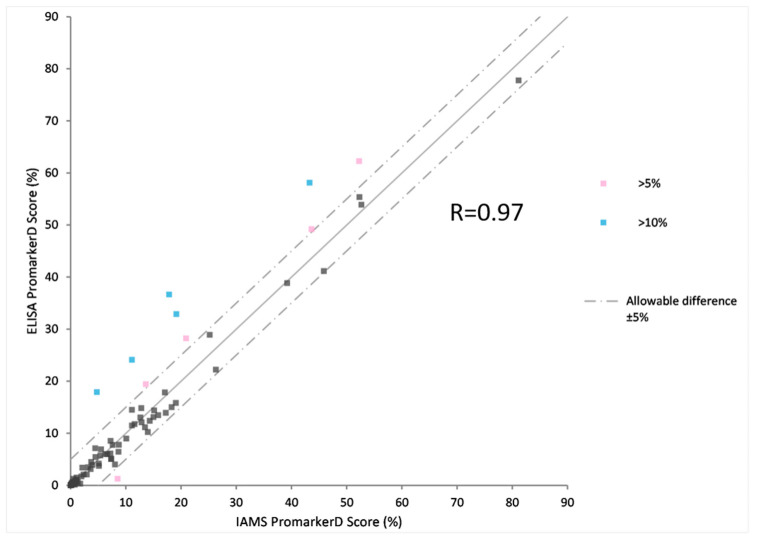
Scatter plot comparing PromarkerD risk predictions based on IAMS and ELISA platforms.

The PromarkerD risk score is a prognostic prediction of a rapid decline in renal function. To prove the utility of the PromarkerD test as either an immunoaffinity (IAMS) or immunocapture (ELISA) method the two platforms were both used to measure biomarker concentrations from 100 plasma samples from the FDS collection. After a Bland Altman analysis of each protein, ELISA biomarker concentrations were adjusted (Figure 2, Figure 3 and Figure 4). The PromarkerD risk score was calculated and the results compared between the two methods, with a coefficient of correlation, R = 0.97 achieved (Figure 5).

## 4. Discussion

When creating an assay to test the efficacy of a biomarker to diagnose a disease state the initial considerations are naturally the practical considerations of accurately measuring the values required. Assay length, reproducibility in other laboratories, stability and clinical aspirations are often not addressed and take a backseat to the research-grade requirements. In the case of the PromarkerD test for chronic kidney disease, these factors have been addressed in this study and the two variations of the initial research method using immunodepletion, namely an immunoaffinity capture method and an ELISA method, are reflective of these considerations.

### 4.1. The “New” IAMS Immunoaffinity LCMSMS Assay

This manuscript has initially described the IAMS method with the conversion from a week-long immunodepletion method from processing to data analysis, into a two-day assay output for a batch of samples. This IAMS method was transformed from a labour intensive and expensive immunodepletion workflow that targeted three biomarkers from within a still complex sub-proteome of plasma, to an elegant multiplexing immunoaffinity capture-based methodology providing a clean and selective sample comprising only the biomarkers of interest. 

An essential component of the viability of a clinical assay is the stability of the analytes under expected or potential conditions that might influence either the processing or detection characteristics. The temperature and time variables tested for this assay demonstrated the stable behaviour of the biomarkers under these conditions. Any assay for a clinical application to be employed over an extended period of time needs to be reproducible, both intra-assay and inter-assay, as demonstrated in this study. 

### 4.2. The ‘Old’ ELISA

The ELISA technology is a tried and trusted method for clinical biomarker measurement and is typically the method of choice for clinical laboratories. To complement the IAMS method and provide options for commercial applications, the PromarkerD test was also converted into an ELISA format. Due to the large protein concentration range across the three biomarkers, it was not possible to multiplex the plasma processing as each biomarker used a different dilution of the plasma. However, a single plasma sample was diluted in series to provide detection of each of the three biomarkers in three separate plates in parallel. The stability of the plasma biomarkers was tested with multiple freeze-thaw cycles showing no significant deviation from the nominal values of a no-freeze thaw experiment. The inter-assay and intra-assay experiments also showed minimal deviation for expected values demonstrating the robustness of the ELISA platform in this application.

### 4.3. The ‘New’ versus the “Old”: IAMS and ELISA

The IAMS and ELISA platforms were tested against each other on a cohort of 100 plasma samples. Comparability of the individual PromarkerD biomarker concentrations were examined and a Bland Altman statistical analysis used to show the biomarker concentration data displayed no statistically significant difference between the two methods. However, differences in the relative performance of the two technologies between proteins were observed, specifically assays for CD5L and IBP3 gave higher correlations in sample concentration measurements than APOA4. These differences in performance merit investigation in future studies. Of note, and contrary to expectations, the ELISA’s exhibited greater dynamic range than the IAMS assays, which also merits further investigation.

The three proteins themselves showed comparable stability to variations in sample storage conditions, including freeze-thaw cycling, across both methods. This is a prerequisite for reproducible sample testing in a clinical environment where shipping conditions need to be flexible.

Individual protein concentration measurements were followed by a comparison of the PromarkerD risk score for progression of diabetic kidney disease for each of the 100 samples. There was high correlation between the “new” IAMS and “old” ELISA platforms with >90% of the cohort having risk scores within 5% of each platform’s score. This demonstrates the ability of either of these technologies to be used as clinical level assays for the measurement of PromarkerD biomarkers. 

### 4.4. Protein Biomarker Assays in the Clinic 

The power of proteomics is leading to the identification of a vast number of biomarkers for numerous diseases, with a Google Scholar search of the terms “proteomics” and “biomarker” yielding 300,000 hits. Despite this number, few proteomic biomarker panels have successfully transitioned from discovery to clinical use [14,15], with limited examples available: OVA2 for ovarian cancer [16], VeriStrat for lung cancer [17], Vectra for rheumatoid arthritis [18].

The major obstacles to this transition remain assay reproducibility and current clinical workflows which preference easy-to-use, low-cost technologies and fast turnaround times. Contrasting this, the omics revolution is highlighting the benefits of measuring multiple biomarkers simultaneously to achieve more accurate diagnoses of the disease state [19,20]. Although ELISA based platforms continue to drive current clinical practice, the assays themselves remain slow to develop, vulnerable to cross-reactivity, and difficult to multiplex. 

The technology comparison described here emphasises that new developments in clinical proteomics, such as IAMS, can be applied effectively and in relatively high throughput to the measurement of multiplex biomarker panels. Importantly the approach uses the same concepts in sample enrichment which have proved so effective and reliable by ELISA, but with more adaptable and precise instrumentation. 

## 5. Conclusions 

Determining the concentration of a protein biomarker accurately, efficiently and precisely, is a requirement for any assay that relies on that measurement in making contributions to clinical decision making. We suggest the successful application of the three-protein PromarkerD test by immunoaffinity mass spectrometry offers a glimpse of the potential of this technique in clinical practice in the decades to come. Although each of the PromarkerD test methods discussed in this manuscript have different approaches, they all can provide a prognostic prediction of diabetic nephropathy outcome and identify at-risk patients allowing earlier intervention and monitoring of disease progression. This test and the technologies to implement it are a useful clinical tool to improve patient outcomes.

## Figures and Tables

**Table 1:** IAMS and ELISA working ranges and dilutions.

Protein	IAMS		ELISA		
	Plasma Working Ranges (µg/mL)	LOD (µg/mL)	Assay Working Ranges (ng/mL)	Plasma Dilution Factor	Plasma Working Ranges (µg/mL)
APOA4	37.5–200	9.40	6.64–425	1 in 400	2.60–170
CD5L	0.104–10.0	0.100	0.16–10	1 in 1600	0.26–16.0
IBP3	0.0104–1.00	0.010	0.63–40	1 in 200	0.13–8.0

**Table 2:** IAMS and ELISA intra-assay and inter-assay variability. Precision based on the average % CV.

Protein	IAMS		ELISA	
	Intra-Assay (*N* = 4) Precision (% CV)	Inter-Assay (*N* = 20) Precision (% CV)	Intra-Assay (*N* = 3) Precision (% CV)	Inter-Assay (*N* = 3) Precision (% CV)
APOA4	9.4	9.4	4.2	2.8
CD5L	7.6	9.8	3.4	3.9
IGFBP3	5.6	10.5	2.7	11.8

## Supplementary Materials

The following are available online at https://www.mdpi.com/2227-7382/8/4/31/s1, Table S1: The baseline demographic and clinical characteristics of the 100 Fremantle Diabetes Study participants included in the current study. Table S2: Transition settings for mass spectrometry analysis.

## Abbreviations

Ab-beads	Ab-beads magnetic beads conjugated with monoclonal antibodies to the biomarker proteins
ACR	Albumin:creatinine ratio
APOA4	Apolipoprotein A4
CD5L	CD5 antigen-like
CKD	Chromic kidney disease
CV	Coefficient of variation
eGFR	Estimated glomerular filtration rate
EDTA	Ethylenediaminetetraacetic acid
ELISA	Enzyme-linked immunosorbent assay
FDA	Food and Drug Administration
FDS	Fremantle Diabetes Study
HSA	Human serum albumin
IAM	Iodoacetamide
IAMS	Immunoaffinity mass spectrometry
IBP3	Insulin-like growth factor-binding protein 3
LCMS	Liquid chromatography mass spectrometry
LCMSMS	Liquid chromatography mass spectrometry/mass spectrometry
LOD	Limits of detection
LOQ	Limit of quantification
MRM	Multiple reaction monitoring
N	Number
NaCl	Sodium chloride
PBS	Phosphate buffered saline
QC	Quality control
R	Coefficient of correlation
R^2^	Coefficient of determination
RT	Room temperature
SDS-PAGE	Sodium dodecyl sulphate-polyacrylamide gel electrophoresis
S/N	Signal to noise
TCEP	Tris(2-carboxyethyl) phosphine hydrochloride
TEAB	Triethylammonium bicarbonate
